# Supplementary material for: Filming ultrafast roaming-mediated isomerization of bismuth triiodide in solution
Source: Nat Commun. 2021 Aug 5;12:4732. doi: 10.1038/s41467-021-25070-z (PMC8342516; doi:10.1038/s41467-021-25070-z)
Supplement: Supplementary file 1 — Supplementary Information [file 41467_2021_25070_MOESM1_ESM.pdf]

Supplementary Information

Filming ultrafast roaming-mediated isomerization of bismuth triiodide in solution

Eun Hyuk Choi[1,2], Jong Goo Kim[1,2], Jungmin Kim[1,2], Hosung Ki[1,2], Yunbeom Lee[1,2], Seonggon Lee[1,2], Kihwan Yoon[3], Joonghan Kim[3], Jeongho Kim[4], and Hyotcherl Ihee[1,2*]

[1]Department of Chemistry and KI for the BioCentury, Korea Advanced Institute of Science and Technology (KAIST), Daejeon 34141, Republic of Korea
[2]Center for Nanomaterials and Chemical Reactions, Institute for Basic Science (IBS), Daejeon 34141, Republic of Korea
[3]Department of Chemistry, The Catholic University of Korea, Bucheon 14662, Republic of Korea
[4]Department of Chemistry, Inha University, Incheon 22212, Republic of Korea

*Correspondence to: hyotcherl.ihee@kaist.ac.kr.

Table of Contents

Supplementary Methods **Pages**

1. **Femtosecond time-resolved x-ray liquidography (fs-TRXL) experiment** ······ S4
2. **Data processing** ······ S4 – S6
3. **Singular value decomposition of $\Delta S_{iso}(q, t)$ and $\Delta S_{aniso}(q, t)$** ······ S6 – S7
4. **Extraction of the difference scattering signal, $\Delta S_{iso}'(q, t)$, representing the exponential kinetics** ······ S7 – S8
5. **Global fit analysis on $\Delta S_{iso}'(q, t)$** ······ S8 – S12
6. **Structural analysis on $\Delta S_{iso}(q, t \geq 175$ fs)** ······ S12 – S14
7. **Structural analysis on $\Delta S_{iso}(q, t < 175$ fs)** ······ S14 – S16
8. **Computational details of quantum and DFT calculations** ······ S16 – S17

Supplementary Notes **Pages**

1. **Supplementary Note 1: Root-mean-squared displacement (σ) for the atomic pairs containing I_c of the early isomer** ······ S17 – S18
2. **Supplementary Note 2: Simulation to check the effect of IRF on structural analysis of $\Delta S_{iso}(q, t < 175$ fs)** ······ S18

References **Pages**

1. **References** ······ S39

Supplementary Figures **Pages**

1. **Supplementary Fig. 1** ········ S19
2. **Supplementary Fig. 2** ········ S20
3. **Supplementary Fig. 3** ········ S21
4. **Supplementary Fig. 4** ········ S22
5. **Supplementary Fig. 5** ········ S23
6. **Supplementary Fig. 6** ········ S24
7. **Supplementary Fig. 7** ········ S25
8. **Supplementary Fig. 8** ········ S26
9. **Supplementary Fig. 9** ········ S27
10. **Supplementary Fig. 10** ········ S28
11. **Supplementary Fig. 11** ········ S29
12. **Supplementary Fig. 12** ········ S30
13. **Supplementary Fig. 13** ········ S31
14. **Supplementary Fig. 14** ········ S32
15. **Supplementary Fig. 15** ········ S33
16. **Supplementary Fig. 16** ········ S34
17. **Supplementary Fig. 17** ········ S35
18. **Supplementary Fig. 18** ········ S36
19. **Supplementary Fig. 19** ········ S37

Supplementary Tables **Pages**

1. **Supplementary Table 1** ········ S38

Supplementary Methods

1. Femtosecond time-resolved x-ray liquidography (fs-TRXL) experiment

The experiment was conducted at the XSS beamline of PAL-XFEL (the Pohang Accelerator Laboratory X-ray Free-Electron Laser)[1,2]. The XFEL delivered x-ray pulses with 12.7 keV energy and a temporal width less than 50 fs at a repetition rate of 30 Hz to the experimental hall of the XSS beamline, and the x-ray beam was focused to a spot of ~30 μm diameter. Optical pump pulses at the wavelength of 400 nm were generated via second-harmonic generation of the 800 nm femtosecond laser pulses from a Ti:sapphire regenerative amplifier. The optical pump pulses were temporally compressed to a temporal width of ~100 fs at the sample position and spatially focused to a spot of ~200 μm diameter, yielding a laser fluence of ~1.8 mJ/mm^2. The x-ray and the optical laser beams were overlapped at the sample position in a collinear geometry.

The sample solution of BiI_3 was prepared by dissolving powder of bismuth iodide (BiI_3, Aldrich, 99%) in acetonitrile (Aldrich, anhydrous, 99.8%) at a concentration of 1 mM. In addition, 0.5 mM a dye, 4-bromo-4′-(N,N-diethylamino)-azobenzene (HANCHEM, 99.9%), in acetonitrile was prepared to measure the solvent heating signals. All of the reagents were used as received without further purifications. The sample solution was circulated through a quartz capillary nozzle, which provided a free-flowing cylindrical liquid jet of 100 μm diameter, delivering a fresh sample solution for every pump-probe pair. Scattered x-rays were detected by a Rayonix MX225-HS CCD (5760 × 5760 pixels with a pixel size of 39 μm) placed ~33.7 mm behind the liquid jet, which allowed a q-space coverage up to ~7.5 $Å^{-1}$. The detector was operated in the 4 × 4 binning mode. The scattering images were measured at 134 pump-probe time delays, which were −950 fs – 1.95 ps (with 25 fs time interval), 2.51 ps, 3.16 ps, 3.98 ps, 5.01 ps, 6.31 ps, 7.94 ps, 10 ps, 12.5 ps, 15.8 ps, 19.9 ps, 25.1 ps, 31.6 ps, 39.8 ps, 501. ps, 63.0 ps, 79.4 ps, and 100 ps. The laser-off images were repeatedly measured at a time delay of −3.0 ps before taking every laser-on image. We performed 13 independent sets of measurements (runs) and obtained ~2,560 images per time delay on average. The timing jitter between the x-ray and optical laser pulses was corrected in real time using an Optical laser and XFEL Cross-correlator (OXC) timing feedback tool.

2. Data processing

The time-resolved two-dimensional (2D) difference scattering images were obtained by subtracting the laser-off images from the laser-on images. The 2D difference scattering images

were converted to one-dimensional (1D) azimuthally-integrated difference scattering curves, $\Delta S_{azimut}(q, t)$, by calculating the average intensity as a function of the momentum transfer, $q = (4\pi/\lambda)\cdot\sin(2\theta/2) = (4\pi/\lambda)\cdot\sin[1/2\cdot\tan^{-1}(l/d)]$, where λ is the wavelength of x-ray, the 2θ is the scattering angle, l is the distance from the beam center to a given pixel and d is the sample-to-detector distance (see Supplementary Fig. 2). The resultant scattering curves are contaminated by systematic noise. To get rid of the systematic noise, we applied the SANOD method[3] to $\Delta S_{azimut}(q, t)$ and used the output $\Delta S_{azimut}(q, t)$ for the subsequent data processing (see Supplementary. Fig. 4).

As can be seen in Supplementary Fig. 2, the raw difference scattering images are anisotropic, and therefore $\Delta S_{azimut}(q, t)$ can be decomposed into anisotropic and isotropic components. Based on a well-established method[4,5], the 2D difference scattering images at each time delay were decomposed into 1D anisotropic and isotropic difference scattering curves, $\Delta S_{aniso}(q, t)$ and $\Delta S_{iso}(q, t)$, respectively (see Supplementary Fig. 4). Briefly, the difference scattering images can be decomposed as follows

$$\Delta S(q,\theta_q,t) \propto \Delta S_{\text{iso}}(q,t) + P_2(\cos\theta_q)\Delta S_{\text{aniso}}(q,t) \quad \text{(S1)}$$

where $P_2(x) = (3x^2 - 1)/2$ is the second-order Legendre polynomial, and θ_q is the angle between the laser polarization axis and the momentum transfer vector. For the configuration of fs-TRXL experiment where x-ray beam and optical laser are almost collinear, which is the standard experimental geometry of fs-TRXL including that of this work, $\cos\theta_q$ can be expressed as follows

$$\cos\theta_q = -\cos\theta\cos\phi \quad \text{(S2)}$$

where ϕ is the azimuthal angle on the detector plane. Following the linear relation between $P_2(\cos\theta_q)$ and the scattering intensity in equation (S1), the contributions of $\Delta S_{aniso}(q, t)$ and $\Delta S_{iso}(q, t)$ can be considered as the slope and intercept, respectively, of a linear function with $P_2(\cos\theta_q)$ as an independent variable defined along the ϕ on the detector plane at a given θ (or q). Therefore, at each q, $\Delta S_{aniso}(q, t)$ and $\Delta S_{iso}(q, t)$ as well as their corresponding errors can be obtained through a linear-regression fit.

The systematic noises generate an artifact in the scattering curve with the shape similar to the scattering curve arising from the solvent density change mainly in the low-q region. Considering that the spatial fluctuation of the liquid jet is one of the major origins of the systematic noises, we can infer that the systematic noises affect the scattering curve in an isotropic manner. Therefore, most of the systematic noises arise from $\Delta S_{iso}(q, t)$ and $\Delta S_{aniso}(q, t)$ should be free from the effect of systematic noises. By taking advantage of the noise-free $\Delta S_{aniso}(q, t)$, we filtered out

the noise from $\Delta S_{iso}(q, t)$ in the following manner. First, 2D anisotropic images were inversely reconstructed from $\Delta S_{aniso}(q, t)$. Then, by azimuthally averaging the reconstructed 2D anisotropic images, we obtained the anisotropic component of the azimuthally averaged 1D difference scattering curves, $\Delta S_{aniso,azimut}(q, t)$ (see Supplementary Fig. 4). By subtracting this anisotropic component from $\Delta S_{azimut}(q, t)$, noise-filtered $\Delta S_{iso}(q, t)$ were obtained (see Supplementary Fig. 4). These procedures were applied to each of multiple runs. After correcting the time-zero of each run and taking an average, we finally obtained $\Delta S_{iso}(q, t)$, which were used in the subsequent analysis. The resultant $\Delta S_{iso}(q, t)$ were further scaled to the absolute scale corresponding to the scattering intensity of one solvent molecule by multiplying the scaling factor, which was obtained from the comparison of the scattering intensity in the high-q region (q = 3.5 ~ 6.5 Å^{-1}) of $\Delta S_{iso}(q, 100\text{ ps})$ with that of the 100-ps scattering curve measured at ESRF, which had been already scaled to the absolute scale in our previous study[6]. In supplementary Fig. 16, the scaled $\Delta S_{iso}(q, 100\text{ ps})$ is shown together with the $\Delta S_{azimut}(q, 100\text{ps})$ measured at ESRF.

3. Singular value decomposition of $\Delta S_{iso}(q, t)$ and $\Delta S_{aniso}(q, t)$

To examine the temporal behavior of the scattering data, we applied the singular value decomposition (SVD) analysis to both $\Delta S_{aniso}(q, t)$ and $\Delta S_{iso}(q, t)$ by reforming each data set into the form of $n_q \times n_t$ matrix, **M**, where n_q and n_t are the number of data points along the q- and t-axes, respectively. Upon the SVD, each of the $\Delta S_{aniso}(q, t)$ and $\Delta S_{iso}(q, t)$ can be decomposed into three matrices satisfying the relationship $\mathbf{M} = \mathbf{U}\cdot\mathbf{S}\cdot\mathbf{V}^{\mathrm{T}}$ where the superscript **T** means the transpose of a matrix, **U** is an $n_q \times n_t$ matrix whose column vectors are called the left singular vectors (LSVs), **S** is a diagonal $n_t \times n_t$ matrix whose elements (s_i, where $i = 1 – n_t$) are called singular values of a matrix **M**, and **V** is an $n_t \times n_t$ matrix whose column vectors are called the right singular vectors (RSVs). The LSVs represent time-independent scattering curves, and RSVs represent the time-dependent amplitude changes of the corresponding LSVs. The singular values represent the weights of the corresponding LSVs and RSVs. Since the singular values are ordered such that $s_1 \geq s_2 \geq \ldots \geq s_n \geq 0$, the left-hand columns of **U** and **V** have a larger contribution to the data.

From the SVD analysis of $\Delta S_{iso}(q, t)$, four significant RSVs were obtained (see Supplementary Fig. 5). The first five RSVs of $\Delta S_{iso}(q, t)$ of the BiI_3 solution weighted by the corresponding singular values are shown in Supplementary Fig. 5a. Among those RSVs, 3rd and 4th RSVs (RSV_3 and RSV_4) mainly contribute to the signal on the sub-ps time scales; when the

two RSVs are removed from $\Delta S_{iso}(q, t)$, the ultrafast shifts of the positive and negative peaks in q-space occurring at < 500 fs disappear, as shown in Supplementary Fig. 17. Therefore, we can infer that the first two RSVs of the $\Delta S_{iso}(q, t)$ of the BiI_3 solution contain the population kinetics of reacting chemical species while RSV_3 and RSV_4 are associated with coherent motions of BiI3. Thus, to extract the population kinetics, we globally fit the first two RSVs using a sum of exponential functions convoluted with a Gaussian instrumental response function (IRF). As shown in Supplementary Fig. 6a and b, a sum of four exponential functions was enough to fit those two RSVs satisfactorily, whereas a sum of three exponential functions did not. As a result, we obtained four time constants, 508 (± 13) fs, 3.11 (± 0.43) ps, 8.83 (± 1.11) ps, and 11.90 (± 1.67) ps, and the full width at half-maximum (FWHM) of the IRF was determined to be 162 (± 7) fs.

In Supplementary Fig. 3, the first two RSVs and LSVs of $\Delta S_{aniso}(q, t)$ are shown in comparison with $\Delta S_{aniso}(q, t)$ of a dye solution, and it can be seen that $\Delta S_{aniso}(q, t)$ are dominated by the first two singular values. We note that, as shown in Supplementary Figs. 3a and b, the first two SVs of significant contributions obtained from $\Delta S_{aniso}(q, t)$ of BiI_3 solution are nearly identical to those obtained from $\Delta S_{aniso}(q, t)$ of a dye solution. Since $\Delta S_{aniso}(q, t)$ of the dye solution originates from the optical Kerr effect (OKE) of neat solvent, that is, transient alignment of solvent molecules induced by polarized optical field, the similarity of $\Delta S_{aniso}(q, t)$'s of the BiI_3 and dye solutions indicates that the structural dynamics of BiI_3, which is of our interest, are not reflected in $\Delta S_{aniso}(q, t)$ but contained only in $\Delta S_{iso}(q, t)$. Therefore, only $\Delta S_{iso}(q, t)$ of the BiI_3 solution was further analyzed.

4. Extraction of the difference scattering signal, ΔS_{iso}'(q, t), representing the exponential kinetics

The difference scattering signal, $\Delta S_{iso}(q, t)$, of BiI_3 solution has rich features including the signals from both exponential kinetics and coherent dynamics. To simplify the analysis, we first extracted and analyzed the contribution corresponding to the exponential kinetics, termed ΔS_{iso}'(q, t). As explained in the previous section, the first two RSVs of the $\Delta S_{iso}(q, t)$ of the BiI_3 solution contain information on the exponential kinetics. To reconstruct ΔS_{iso}'(q, t), the 1st and 2nd RSVs in the **V** of $\Delta S_{iso}(q, t)$ were replaced by their corresponding fit curves, and the 3rd and 4th SVs were filtered by setting the corresponding singular values, LSVs and RSVs to zero in the **S**, **U** and **V**, generating a new set of matrices, **U'**, **S'**, and **V'**. By using the relation $\mathbf{M} = \mathbf{U}\cdot\mathbf{S}\cdot\mathbf{V}^T$, ΔS_{iso}'(q, t) were

constructed in the form of **U'·S'·V'**^{T}. These procedures are relevant only for the scattering data at early time delays, where coherent dynamics are present, and therefore were applied to the data up to the time delay of 2 ps. Since the coherent dynamics are nearly absent after 2 ps in our data, $\Delta S_{iso}'(q, t)$ was set to be equal to the original data, $\Delta S_{iso}(q, t)$, after 2 ps. The reconstructed signals, $\Delta S_{iso}'(q, t)$, representing the exponential kinetics are shown in Supplementary Fig. 18. As stated in the previous section, $\Delta S_{iso}(q, t)$ of BiI_3 solution was fitted by four exponentials with time constants, 508 (± 13) fs, 3.11 (± 0.43) ps, 8.83 (± 1.11) ps, and 11.90 (± 1.67) ps,

The separation of signal contributions corresponding to exponential kinetics and coherent dynamics can be expressed as follows:

$$\Delta S(q,t) = c(t) * \{S^{\text{es}}(q,t) - S^{\text{gs}}(q)\} + \Delta S_{\text{solvent}}(q,t) \quad \text{(S3)}$$

$$= c(t) * [\{S^{\text{es}}(q,t) - S^{\text{es,eq}}(q)\} + \{S^{\text{es,eq}}(q) - S^{\text{gs}}(q)\}] + \Delta S_{\text{solvent}}(q,t) \quad \text{(S4)}$$

$$= \left[c(t) * \{S^{\text{es}}(q,t) - S^{\text{es,eq}}(q)\}\right] + \left[c(t) * \{S^{\text{es,eq}}(q) - S^{\text{gs}}(q)\} + \Delta S_{\text{solvent}}(q,t)\right] \quad \text{(S5)}$$

$$= \Delta S_{\text{residue}}(q,t) + \Delta S^{'}(q,t) \quad \text{(S6)}$$

where $c(t)$ is the fractional concentration of excited molecules, $S^{es}(q\ ,t)$, $S^{es,eq}(q)$, $S^{gs}(q)$ is the scattering signals from the time-dependent structures of the solute (or cage) in the excited-state, equilibrium structure of the solute (or cage) in the excited-state and the structure of the solute (or cage) in the ground-state, respectively, and $\Delta S_{solvent}(q, t)$ is the difference scattering signal arising from the temperature rise of the bulk solvent, whose detailed description is given in the following section. Therefore, the kinetics of solute and solvent as well as time-independent excited-state structures can be obtained by analyzing $\Delta S^{'}(q, t)$. Then, as the next step, the time-dependent snapshots of the structures of reacting molecules can be obtained by analyzing $\Delta S_{residue}(q, t)$ or, equivalently, the whole signal, $\Delta S(q, t)$, based on the information of kinetics and equilibrium structure obtained from $\Delta S'(q, t)$. In this work, we chose the later method.

5. Global fit analysis on $\Delta S_{iso}'(q, t)$

As mentioned in the main text, the previous TRXL study on BiI_3 in solution reported an isomer (*iso*-BiI_2-I) and dissociation fragments (BiI_2· and I·) are the only detectable solute species at 100 ps [6]. Fitting $\Delta S_{iso}'(q, t)$ at earlier time delays, for example, 1 ps, using the scattering curves of these known components of solute and solvent did not provide any satisfactory agreement, as shown in

Supplementary Fig. 8. Therefore, we hypothesized that an additional intermediate, "X", was involved in the time delays earlier than 100 ps.

Then, we built kinetic models including the X intermediate by assuming that three out of the four time constants obtained by fitting RSVs correspond to the kinetics of solute and the other one to the heating kinetics of bulk solvent. According to our previous result [6], "X" should completely decay to another species before 100 ps. Also, the fs-TRXL data of the BiI_3 solution shown in Fig. 2a undergoes a significant reduction of the overall amplitude over time. Thus, at least one of the reacting species should relax back to the ground-state BiI_3. We built eleven kinetic model frames compatible with these conditions, as shown in Supplementary Fig. 9. These eleven kinetic model frames require three time constants, τ_a, τ_b, and τ_c, to be assigned, as shown in Supplementary Fig. 9. Accordingly, three time constants from the four time constants obtained from the fit of RSVs were selected and distributed to τ_a, τ_b, and τ_c while the remaining time constant was assigned to the solvent heating. In this way, we built a total of 264 kinetic models (24 per each kinetic model frame), each of which was tested through global fit analysis (GFA) on $\Delta S_{iso}'(q, t)$. We note that if only three exponentials are used, then the whole kinetic framework of the solute needs to be described with only two exponentials (one is for the solvent heating). Such a kinetic framework cannot explain the experimental data well.

In GFA, $\Delta S_{iso}'(q, t)$ was fit against theoretical scattering curves, $\Delta S_{theory}'(q, t)$, by minimizing the reduced χ^2 value (χ^2_{red}), which is defined as follows

$$\chi^2_{red} = \frac{1}{N-p-1} \sum_{j=\text{time delay}} \sum_{i} \frac{\left(\Delta S_{theory}'(q_i, t_j) - \Delta S_{iso}'(q_i, t_j)\right)^2}{\sigma^2_{i,j}} \tag{S7}$$

where N is the total number of data points along the q- and t-axes, p is the number of fit parameters, and $\sigma_{i,j}$ is the standard deviation of the difference scattering intensity at i^{th} q of jth time delay. The χ^2_{red} minimization was performed using the MINUIT package written at CERN and the error analysis was performed by MINOS, a built-in algorithm in the MINUIT software[7]. $\Delta S_{theory}'(q, t)$ was constructed by a linear combination of the scattering signals of solute and solvent as follows [8]

$$\Delta S_{theory}'(q,t) = \Delta S_{solute}(q,t) + \Delta S_{cage}(q,t) + \Delta S_{solvent}(q,t) \tag{S8}$$

where the symbols are explained in the following. During the GFA process, (i) the excitation ratio, (ii) a prefactor for the exponential growth function for describing the temperature rise of the solvent, and (iii) the structural parameters for "X", which will be described in the following section,

were used as the free parameters for the fit. Depending on the kinetic model frame, several branching ratios are used as free parameters among eight branching ratios, which are (i) the ratio between the initial "X" and the dissociation fragments, (ii) the ratio of "X" relaxing to the *iso*-BiI_2-I, (iii) the ratio of "X" relaxing to the ground-state BiI_3, (iv) the ratio of "X" relaxing to the dissociation fragments, (v) the ratio of dissociation fragments recombining to the *iso*-BiI_2-I, (vi) the ratio of dissociation fragments recombining to the ground-state BiI_3, (vii) the ratio of dissociation fragments recombining to "X", and (viii) the ratio of the remaining dissociation fragments. For model frame A, four branching ratios of (i), (ii), and (vi) are used as free parameters, while the ratio of the remaining dissociation fragments and the ratio of "X" relaxing to the ground-state BiI_3 are determined as 1−branching ratio (vi) and 1−branching ratio (ii), respectively. For model frame B, four branching ratios of (i), (v), (vi) and (viii) are used as free parameters. For model frame C, three branching ratios of (i), (ii), and (v) are used as free parameters, while the ratio of the remaining dissociation fragments and the ratio of "X" relaxing to the ground-state BiI_3 are determined as 1−branching ratio (v) and 1−branching ratio (ii), respectively. For model frame D, four branching ratios of (i), (v), (vi), and (viii) are used as free parameters. For model frame E, four branching ratios of (i), (v), (vi), and (viii) are used as free parameters. For model frame F, three branching ratios of (i), (iv), and (v) are used as free parameters, while the remaining dissociation fragments and the ratio of "X" relaxing to the ground-state BiI3 are determined as 1 −branching ratio (v) and 1−branching ratio (iv), respectively. For model frame G, three branching ratios of (i), (iv), and (v), while the ratio of "X" relaxing to the *iso*-BiI_2-I are determined as 1− branching ratio (v) and 1−branching ratio (iv), respectively. For model frame H, three branching ratios of (i), (ii), and (iii) are used as free parameters. For model frame I, four branching ratios of (i), (v), (vii), and (viii) are used as free parameters. For model frame J, four branching ratio of (i), (vi), (vii), and (viii) are used as free parameters. For model frame K, three branching ratios of (i), (ii), and (vii) are used as free parameters, while the ratio of the remaining fragments and the ratio of "X" relaxing to the ground-state BiI_3 are determined as 1−branching ratio (vii) and 1− branching ratio (ii), respectively.

The scattering signal of solute, $\Delta S_{solute}(q, t)$, came from the molecular structures of the solute molecules involved in the photoreaction and was calculated by the well-known Debye equation based on the structures of solute molecules. The structures of $BiI_2\cdot$, *iso*-BiI_2-I, and ground-state

BiI_3 were used as reported in the previous TRXL study on the BiI_3 in solution [6]. The structure of "X" was optimized during GFA by using the structural parameters for "X" as free parameters for the fit and using the structure of the ground state BiI_3 as the initial starting structure. To determine the structure of "X", we introduced total five structural parameters, one for simultaneously modifying the two Bi-I bonds (I_a-Bi and Bi-I_b), constraining the two bonds to have the same length, and the others for the Bi-I_b-I_c angle, the I_a-Bi-I_b-I_c dihedral angle, the I_a-Bi-I_b angle, and the $I_b \cdots I_c$ distance.

The scattering signal of the cage, $\Delta S_{cage}(q, t)$, stems from the atomic pair distances between solute and solvent molecules and can be theoretically calculated from pair distribution functions (PDFs) obtained from the molecular dynamics (MD) simulations. Since we have already calculated the cage signal of the dissociation fragments, *iso*-BiI_2-I, and ground-state BiI_3, only the cage signal for "X" was newly calculated. The molecular structure of "X" used to calculate its cage signal was roughly determined by performing GFA by using the cage signal of *iso*-BiI_2-I for that of "X". Then, the obtained structure was used to perform the MD simulation using the MOLDY [9] program with the same condition described in our previous study[6] and obtained PDFs from which the cage signal of "X" was calculated. During the MD simulation, the atomic charges for "X" were approximated to those of ground-state BiI_3. Considering the relatively small contribution of the cage signal compared to that of the solute signal due to the heavy atoms consisting of solute molecules, the discrepancy coming from those approximations should not be significant. We calculated the cage signal of "X" using the charge values obtained from DFT calculations described in Supplementary Method 8, and the calculated cage signal does not show significant difference from that calculated with the charge values of the ground-state BiI_3.

The scattering signal of solvent, $\Delta S_{solvent}(q, t)$, originates from the change of the scattering signal due to a temperature increase of the bulk solvent, $(\partial S(q)/\partial T)_\rho$, scaled by the amount of the temperature change, $\Delta T(t)$. The temperature derivative of the scattering signal, $(\partial S(q)/\partial T)_\rho$, can be obtained from a separate TRXL experiment on a dye solution as described elsewhere[10,11]. The temperature change, $\Delta T(t)$, of the bulk solvent was modeled using an exponential growth function as follows

$$\Delta T(t) = A \cdot \left[1 - \exp\left\{-(t - t_0)/\tau\right\}\right] \cdot H(t - t_0) \tag{S9}$$

where A is a prefactor, t_0 is the time-zero, τ is the time constant, $H(t - t_0)$ is the Heaviside step function. In fact, the temperature of the solvent will also respond to the transition between

intermediates (for example, early isomer → late isomer or early isomer → ground state) because the energy difference between the states is transferred to the solvent, increasing the solvent temperature. Considering the current sample concentration (1 mM), excitation ratio, and the relative energies of the reacting species, a relatively small amount of additional rise of the solvent temperature (~0.05 K for the best kinetic model) is expected. In the fit, however, we did not take this additional source of the temperature change into account because the fit qualities were not affected meaningfully due to the relatively small contribution of the solvent heating signal.

By putting all these components together, ΔS_{theory}'(q, t) in GFA is constructed according to the following equations

$$\begin{aligned}\Delta S_{\text{theory}}{}'(q,t) &= \Delta S_{\text{solute}}(q,t)+\Delta S_{\text{cage}}(q,t)+\Delta S_{\text{solvent}}(q,t)\\ &= \Delta S_{\text{solute-related}}(q,t)+\Delta S_{\text{solvent}}(q,t)\\ &= \frac{1}{R}\left[\sum_k S_k(q)\{c_k(t)\otimes \mathrm{IRF}(t)\}-S_{\mathrm{g}}(q)\sum_k c_k(0)\right]+\left(\frac{\partial S}{\partial T}\right)_\rho\{\Delta T(t)\otimes \mathrm{IRF}(t)\}\end{aligned} \quad \text{(S10)}$$

where R is the number ratio of the solvent molecules to solute molecules, k is the index of the solute species, $c_k(t)$ is the fractional concentration of kth species at time delay t, $S_k(q)$ is the solute-related scattering curve of kth species, which consists of the scattering curves of solute and cage, $S_{\text{g}}(q)$ is the scattering curve of the ground state BiI_3 molecule, IRF(t) is the Gaussian IRF determined in the fitting of RSVs and ⊗ is the convolution operator. The fractional concentration of each species, $c_k(t)$, was calculated by numerically solving differential equations derived from the kinetic models.

In Supplementary Data 1, we summarize the relative χ^2_{red} values with respect to the minimum χ^2_{red} value obtained from GFA on 264 kinetic models. The kinetic model A3 gives the best quality of fit, and the detailed kinetic parameters determined based on this kinetic model are described in the main text and shown in Fig 3a. The concentration profiles of the reacting solute species and the time profile of the temperature rise are shown in Fig. 3b and Supplementary Fig. 10, respectively. The optimized structure of "X" (the early isomer) from GFA based on the kinetic model A3 is shown in Fig. 4.

6. Structural analysis on $\Delta S_{\text{iso}}(q, t \geq 175\text{ fs})$

Based on the kinetics and the equilibrium structures of the intermediates determined in the GFA on ΔS_{iso}'(q, t), the coherent structural motions of the reacting molecules were analyzed by fitting

the $\Delta S_{\text{iso}}(q, t)$. For the time delays later than the experimental IRF ($t \geq 175$ fs), the fit was performed at each time delay independently using the following equations

$$\Delta S_{\text{theory}}(q, t \geq 175 \text{ fs}) = \frac{1}{R}\left[\sum_k S_k(q,t)c_k(t) - S_{\text{g}}(q)\sum_k c_k(0)\right] + \left(\frac{\partial S}{\partial T}\right)_\rho \Delta T(t) \quad \text{(S11)}$$

$$\chi^2_{\text{red}}(t \geq 175 \text{ fs}) = \frac{1}{N_q - p - 1}\sum_i \frac{\left(\Delta S_{\text{theory}}(q_i,t) - \Delta S_{\text{iso}}(q_i,t)\right)^2}{\sigma(q_i,t)^2} \quad \text{(S12)}$$

where N_q is the number of q points, p is the number of parameters, $\sigma(q_i, t)$ is the standard deviation of the ΔS_{iso} at q_i of time delay t. Terms in Equation (S11) are the same as those in Equation (S10) except $S_k(q, t)$. In contrast with the $S_k(q)$ in Equation (S10), the time-dependent structures were taken into account in calculating the Debye equation for the solute signal of the solute-related scattering curve of kth species, $S_k(q, t)$, which is, therefore, time-dependent here. During the fitting, only the structural parameters of “X” and the dissociating BiI_3 were used as free parameters while the parameters for the kinetics of solute and solvent, $c_k(t)$ and $\Delta T(t)$, were fixed to the values determined in the GFA of ΔS_{iso}’(q, t). For the structure of “X”, five structural parameters, one for the two bond lengths, I_a-Bi and Bi-I_b, and the others for Bi-I_b-I_c angle, I_a-Bi-I_b-I_c dihedral angle, I_a-Bi-I_b angle, and I_b···I_c distance, were used. For the dissociating BiI_3, three structural parameters, one for Bi···I_c distance, another for the two bond lengths, I_a-Bi and Bi-I_b, and the other for I_a-Bi-I_b angle. Besides, a Debye-Waller factor (DWF), $e^{\sigma^2 q^2/2}$, was applied only for the atomic pairs accompanying the departing iodine atom, I_c, of the dissociating BiI_3 to compensate for the effects of the wavepacket dispersion during the dissociation. Hence, the root-mean-squared distance, σ, of the DWF was used as another free parameter. Since the dissociating iodine atom has little effect on the theoretical scattering curves at time delays later than 600 fs in the measured q-range (1.0 Å$^{-1} \leq q \leq 6.5$ Å^{-1}), the fully dissociated fragments, BiI_2· and I·, in their equilibrium structures were used instead of the dissociating BiI_3 for the signals at time delays later than 600 fs. An averaged set of structural parameters was obtained by fitting the signals using various initial guesses for the structural parameters at each time delay. Specifically, a random structural pool consisting of ~5000 structures for each time delay was generated within boundary conditions, which are, for the “X”, 2.68 Å ≤ I_a-Bi and Bi-I_b ≤ 2.816 Å, 40° ≤ Bi-I_b-I_c angle ≤ 80°, 55° ≤ I_a-Bi-I_b-I_c dihedral angle ≤ 110°, 90° ≤ I_a-Bi-I_b angle ≤ 100°, 3.0 Å ≤ I_b···I_c ≤ 4.26 Å, and, for the dissociating BiI_3, 2.816 Å ≤ Bi···I_c ≤ 8.0 Å, 2.68 Å ≤ I_a-Bi and Bi-I_b ≤ 2.816 Å, 90° ≤ I_a-Bi-I_b angle ≤ 100°. The boundary conditions were chosen to search the structures between the ground state BiI_3 and the final

equilibrium structure for each species, which is "X" and the dissociation fragments, $BiI_2\cdot$ and $I\cdot$. Every structure in the structural pool of each time delay was used as the initial guess to fit the signal at the given time delay. During the fit, those structures were optimized and the corresponding χ^2_{red} was obtained. With respect to the minimum value of $\chi_{red}{}^2$, min[χ^2_{red} (t)], at the given time delay among the obtained χ^2_{red} (t), the output structures giving less than 1.00001 times the min[χ^2_{red} (t)] were collected and the structural parameters of those structures were averaged. By performing this process at each time delay independently, the time-dependent structural motions were obtained.

7. Structural analysis on $\Delta S_{iso}(q, t < 175$ fs)

For the time delays shorter than, or nearby, the experimental IRF (t < 175 fs), an approach slightly modified from that used for the time delays larger than the experimental IRF was used because directly fitting the difference scattering signals at the time delays shorter than, or nearby, the temporal width of IRF results in distorted structural information as explained in Supplementary Note 2.

Each of the time-dependent parameters for the structures of "X" and dissociating BiI_3 and σ of the DWF was modeled by a quartic polynomial function,

$$x(t) = \sum_{k=0}^{4} a_{4-k} t^{4-k} \quad \text{(S13)}$$

where a_{4-k} is the coefficient of the polynomial function. We note that structural fit with a cubic polynomial degrades the quality of fit while using quintic polynomial results in a similar quality of fit to that with a quartic polynomial. The Debye equations for the solute signal in $S_k(q, t)$ were calculated based on $x(t)$ from which the molecular structures were constructed and the DWF for the dissociating BiI_3 was calculated. Subsequently, the instantaneous theoretical difference scattering curves, $\Delta S_{inst}(q, t)$, were calculated using the following equation

$$\Delta S_{inst}(q,t) = \frac{1}{R}\left[\sum_k S_k(q,t)c_k(t) - S_g(q)\sum_k c_k(0)\right] + \left(\frac{\partial S}{\partial T}\right)_\rho \Delta T(t) \quad \text{(S14)}$$

where the terms in Equation (S14) are the same as those in Equations (S10) and (S11). Then, $\Delta S_{inst}(q, t)$ was temporally blurred by convoluting with the Gaussian IRF, IRF(t), which was determined in the fitting of RSVs, resulting in $\Delta S_{theory}(q, t < 175$ fs) as shown in the following equation

$$\Delta S_{\text{theory}}(q, t < 175\text{ fs}) = \Delta S_{\text{inst}}(q, t') \otimes \text{IRF}(t') \tag{S15}$$

where t' is the dummy variable of time delay. Under the constraints where the polynomial functions smoothly connect the structure at 0 fs, which is the structure of the BiI_3 at Frank-Condon state, and the structures at 175 fs obtained from the fitting using Equations (S10) and (S11), the coefficients of the polynomial functions for each structural parameter were optimized by minimizing χ^2_{red} using the following equation

$$\chi^2_{\text{red}} = \frac{1}{N-p-1} \sum_{j=\text{time delay}} \sum_i \frac{\left(\Delta S_{\text{theory}}(q_i, t_j) - \Delta S_{\text{iso}}(q_i, t_j)\right)^2}{\sigma^2_{i,j}} \tag{S16}$$

where N is the total number of data points along the q- and t-axes, p is the number of fit parameters, and $\sigma_{i,j}$ is the standard deviation of the difference scattering intensity at i^{th} q of jth time delay. The structural parameters obtained in this way show oscillatory features in terms of time. Such oscillations cannot be justified with the current IRF and signal-to-noise ratio even if structural parameters indeed have temporal oscillations. We suspected that the temporal oscillations of structural parameters are artifact caused by use of a polynomial function. To remove the artifact, we applied penalties to parameters that inverted the sign of the first derivative. We minimized a sum of following penalty term (P) and χ^2_{red} instead of χ^2_{red} alone.

$$P = \alpha \cdot \sum_{i \in C} \left(\left| \sum_j \left(\frac{(\partial x_i / \partial t)_{t=t_j}}{\max[(\partial x_i / \partial t)]} \right) \right|^{-1} \right) \tag{S17}$$

where α, which is set to be 100, is a constant weighting factor, x_i is the i-th structural parameter belonging to the subset C in which the sign of the first derivative of the parameter changes, t_j is the j-th time delay, $(\partial x_i/\partial t)_{t = t_j}$ is the value at $t = t_j$ of the first derivative of x_i with respect to t, and $\max[(\partial x_i/\partial t)]$ is the maximum value of the first derivative of x_i with respect to t. The value of this penalty term increases when the temporal profiles of structural parameters have more oscillatory features. Consequently, the addition of the penalty term smooths the resulting temporal profiles of structural parameters.

Finally, the resultant $\Delta S_{\text{theory}}(q, t < 175\text{ fs})$ was concatenated with $\Delta S_{\text{theory}}(q, t \geq 175\text{ fs})$, giving rise to ΔS_{theory} as shown in Fig. 2b, Supplementary Fig. 7 and Supplementary Fig. 18. The optimized $x(t)$ values were concatenated with the corresponding parameters which were used to calculate the solute signal of $\Delta S_{\text{theory}}(q, t \geq 175\text{ fs})$ and are represented in Fig. 5 and Supplementary Fig. 12. The standard deviations for the structural parameters and σ of the DWF at $t < 175$ fs were

calculated based on the covariance matrix, which was calculated during the fit of the coefficients of quartic polynomials using the following equation

$$\sigma_{x(t)}^2 = \sqrt{\sum_{k=0}^{4}(t^{4-k})^2 \mathrm{Var}(a_{4-k}) + 2\sum_{i=0}^{4}\sum_{j=i+1}^{4} t^{4-i} t^{4-j} \mathrm{Cov}(a_{4-i}, a_{4-j})} \quad \text{(S18)}$$

where $\sigma_{x(t)}$ is the standard deviation of the parameter x at time delay t, Var(a_{4-k}) is the variance of the coefficient a_{4-k} and Cov(a_{4-i}, a_{4-j}) is the covariance between the coefficients a_{4-i} and a_{4-j}. We note that, in most cases, the standard deviations obtained at $t < 175$ fs are relatively small compared to those of at $t \geq 175$ fs because of the involvement of additional penalty term as well as the globally constrained parameters over several time delays, rather than a specific time delay, for the analysis at $t < 175$ fs.

8. Computational details of quantum and DFT calculations

The geometry optimization and subsequent harmonic vibrational frequency calculation of BiI_3 (C_{3v}) using the coupled-cluster singles and doubles with perturbative triples (CCSD(T)). The scalar relativistic effects of Bi and I were treated using the relativistic effective core potential (RECP). The triple-ζ basis sets (aug-cc-pVTZ) were used for the valence electrons of Bi and I. This combination is denoted as AVTZ. The solvent (acetonitrile) effect was considered using the integral equation formalism polarizable continuum model (IEFPCM).

The multi-state complete active space perturbation theory second-order (MS-CASPT2) calculations were performed on the optimized structure of BiI_3 using CCSD(T)/AVTZ. The ANO-RCC-VTZP all-electron basis sets were used for Bi and I. The scalar relativistic effects were treated using the Douglas-Kroll-Hess second-order (DKH2) approach. The conductor-like PCM (CPCM) model was used for the solvent effect. The state-average CAS self-consistent field (SA-CASSCF) method was used for the reference wave functions of BiI_3. The active orbitals for the SA-CASSCF calculations consist of 6*p* and 5*p* orbitals of Bi and I, respectively. Therefore, 18 electrons were distributed in 12 orbitals, CAS(18,12). The 7 singlet and 9 triplet states were averaged in the SA-CAS(18,12) calculations. The spin-orbit coupling (SOC) was considered using the state-interaction approach with the MS-CASPT2 calculations. According to the results of MS-CASPT2+SOC calculations (see Supplementary Table 1), we can conclude that the T_1 state of BiI_3 (C_{3V}) is directly populated on the Franck-Condon (FC) region from 400 nm irradiation in the experiment. Therefore, the intrinsic reaction coordinate (IRC) method for the T_1 state of BiI_3 (C_{3V})

was used to examine the relaxation pathway from the FC region. The IRC calculations were performed using CAM-B3LYP with the empirical dispersion correction (D3BJ, Grimme's dispersion with Becke-Johnson damping). The solvent effect was also considered using the IEFPCM method as in the CCSD(T) calculations. In the CAM-B3LYP-D3BJ calculations, the dhf-TZVPP basis sets were used for both Bi and I atoms. The dhf-TZVPP consists of RECPs which is the same as those of aug-cc-pVTZ-pp for Bi and I and segment contracted triple-ζ basis sets for valence electrons of Bi and I. The geometry optimization of the T_1 state of early isomer of BiI_3 was performed using CAM-B3LYP-D3BJ/dhf-TZVPP from the final structure of IRC calculations. All DFT and CCSD(T) calculations were performed using the Gaussian16 program and MS-CASPT2 with SOC calculations were performed using the Molcas8.0 program.

Supplementary Notes

Supplementary Note 1: Root-mean-squared displacement (σ) for the atomic pairs containing I_c of the early isomer

To check the possible effect of σ on the structure of the early isomer, we also analyzed the data by including DWFs on the structure of the early isomer. Since the position of partially dissociated iodine, I_c, is expected to have a distribution, we applied DWFs with a shared σ to the atomic pairs containing I(c) of the early isomer. Following the same procedures introduced in section 3 but including the σ for the early isomer as another free-parameter, we performed global fit analysis on $\Delta S_{iso}'(q, t)$. The fitting results show that the value of σ converges to zero, giving the same kinetics and equilibrium structure shown in Fig. 4. Thus, the equilibrium structure of the early isomer has a fairly well-defined structure within our error ranges of structural parameters. To further check the effect of σ on the early time structural motions of the early isomer, we also performed structure refinement on $\Delta S_{iso}(q, t)$ up to 600 fs, following the procedures in section 4 with including DWFs with a shared σ to the atomic pairs containing I_c of the early isomer. The fit results at each time delay provide σ values varying around 0.1 Å. This value is comparable to the error range for the structural parameters that can modulate those atomic pairs. The Bi-I_b-I_c angle, for example, has an error range of about ±3 degrees. When the Bi-I_b-I_c angle is changed by 3 degrees, the pair distances containing I_c are altered by ~0.14 Å, which covers comparably wider distance distributions to those generated based on the obtained σ.

Thus, the distributions in pair distances during the roaming reaction of BiI_3 might be wide, but they are in the comparable range with the structural error range of our signal. Therefore, we conclude that the effect of σ on the structural dynamics of the early isomer is negligible under the structural and temporal resolution of our experiment.

Supplementary Note 2: Simulation to check the effect of IRF on structural analysis of $\Delta S_{iso}(q, t < 175$ fs)

Directly fitting the difference scattering signals at the time delays shorter than the temporal width of IRF results in distorted structural information, as shown in Supplementary Fig. 19. In Supplementary Fig. 19, we generated mock data of a virtual diatomic molecule whose bond length undergoes an elongation from 2.816 Å to 3.616 Å following a sum of two exponential growth functions whose time constants are 50 fs and 150 fs, respectively. The mock data of instantaneous signals, $q\Delta S_{inst}$, are shown in Supplementary Fig. 19a and are convoluted with a Gaussian IRF whose FWHM is 162 fs to make the temporally blurred signals, $q\Delta S_{conv}$ shown in Supplementary Fig. 19b. The $q\Delta S_{conv}$ was directly fit using the Debye equation at each time delay independently and the fit result is shown in Supplementary Fig. 19c. This mode of data analysis corresponds to the one used to fit the data of $\Delta S_{iso}(q, t \geq 175$ fs). The resultant bond length, $r_{fit}(t)$ is shown in Supplementary Fig. 19d and is compared to the true bond length, $r_{true}(t)$, used for generating the mock data. While the general trends of the bond elongation are captured by the fit, the $r_{fit}(t)$ do not correctly reproduce neither the $r_{true}(t)$ nor the IRF convoluted $r_{true}(t)$. In particular, $r_{fit}(t)$ shows a significant discrepancy at the time delays shorter than the temporal width of the IRF. In Supplementary Fig. 19e, Bi-I_b-I_c angle I_a-Bi-I_b-I_c dihedral angle, which are obtained from fitting ΔS_{iso} using the same analysis method used for fitting $\Delta S_{iso}(q, t \geq 175$ fs), are shown as an example. The two angles evolve in a similar fashion to those shown in Fig. 5a, but the temporal profiles in Supplementary Fig. 19e are distorted as in the case of the mock data.

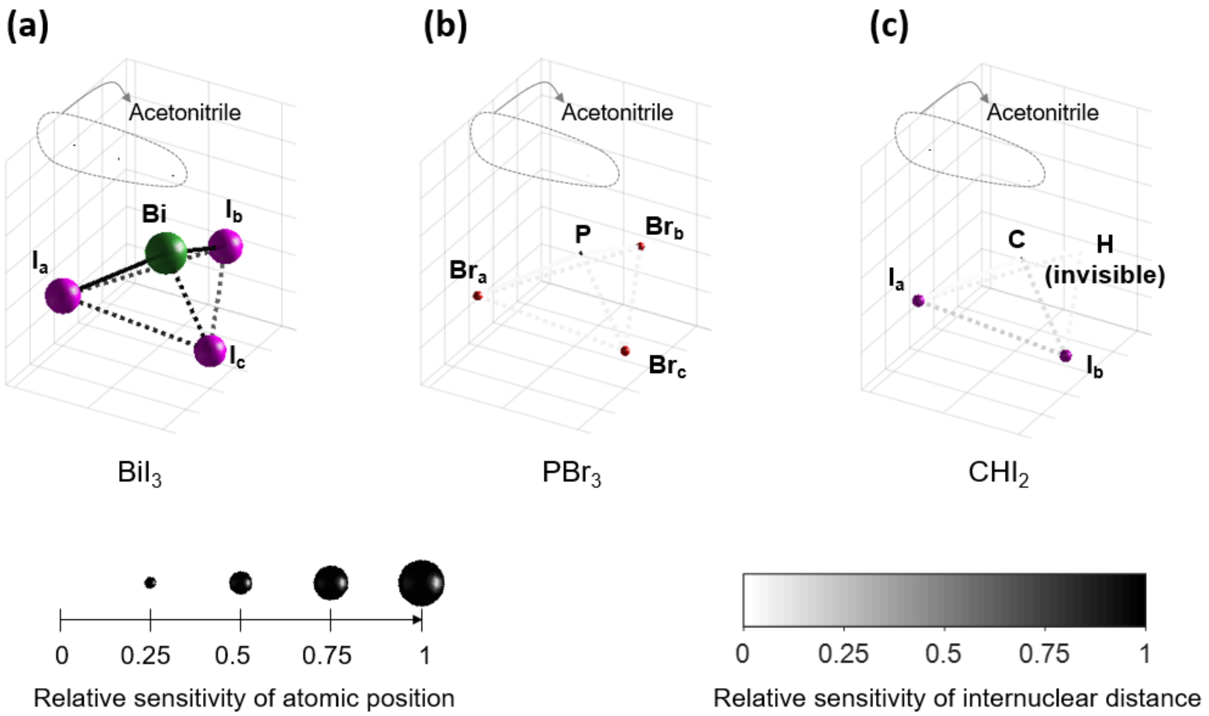

Supplementary Fig. 1. Sensitivity maps for the atomic positions of (a) BiI_3, (b) PBr_3, and (c) CHI_2. The TRXL signal is more sensitive to the position of the atom with the larger sphere. The sensitivity of the solute-solvent cage term is shown by the spheres surrounded by the dotted circle and labeled with "Acetonitrile". Each sensitivity map is obtained by evaluating the change of the TRXL signal upon altering the position of each atom constituting the molecule. The size of the sphere is normalized by that of the Bi atom of BiI_3, which is the most sensitive atom among the atoms in the three molecules shown here. Also, the thickness of the line is normalized by that of $Bi\cdots I_c$ pair of BiI_3, which is the most sensitive pair distances among the atomic pair distances in the three molecules shown here. The TRXL signals were simulated by assuming a ground state molecule having C_{3v} symmetry and an excited state molecule with the identical structure to the early isomer of BiI_3 in Fig. 3c. Thus, except the structure of BiI_3, those of other molecules are artificially devised for the purpose of a fair comparison. This figure visualizes that (i) the relative sensitivity to BiI_3 is drastically larger than to other molecules and (ii) the contribution from the solute-solvent cage term is negligible for all cases.

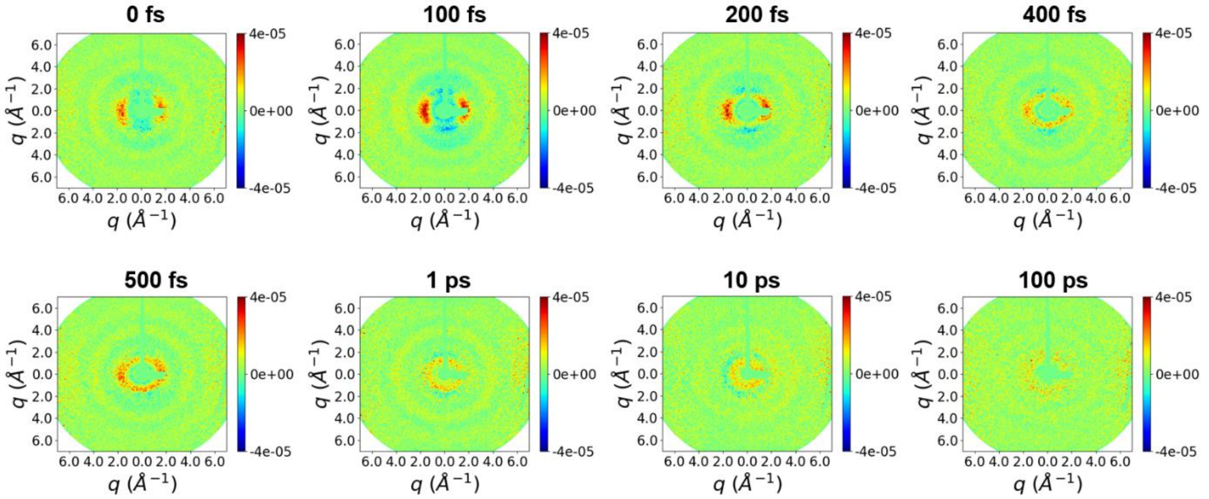

Supplementary Fig. 2. Raw 2D difference scattering images at representative time delays.

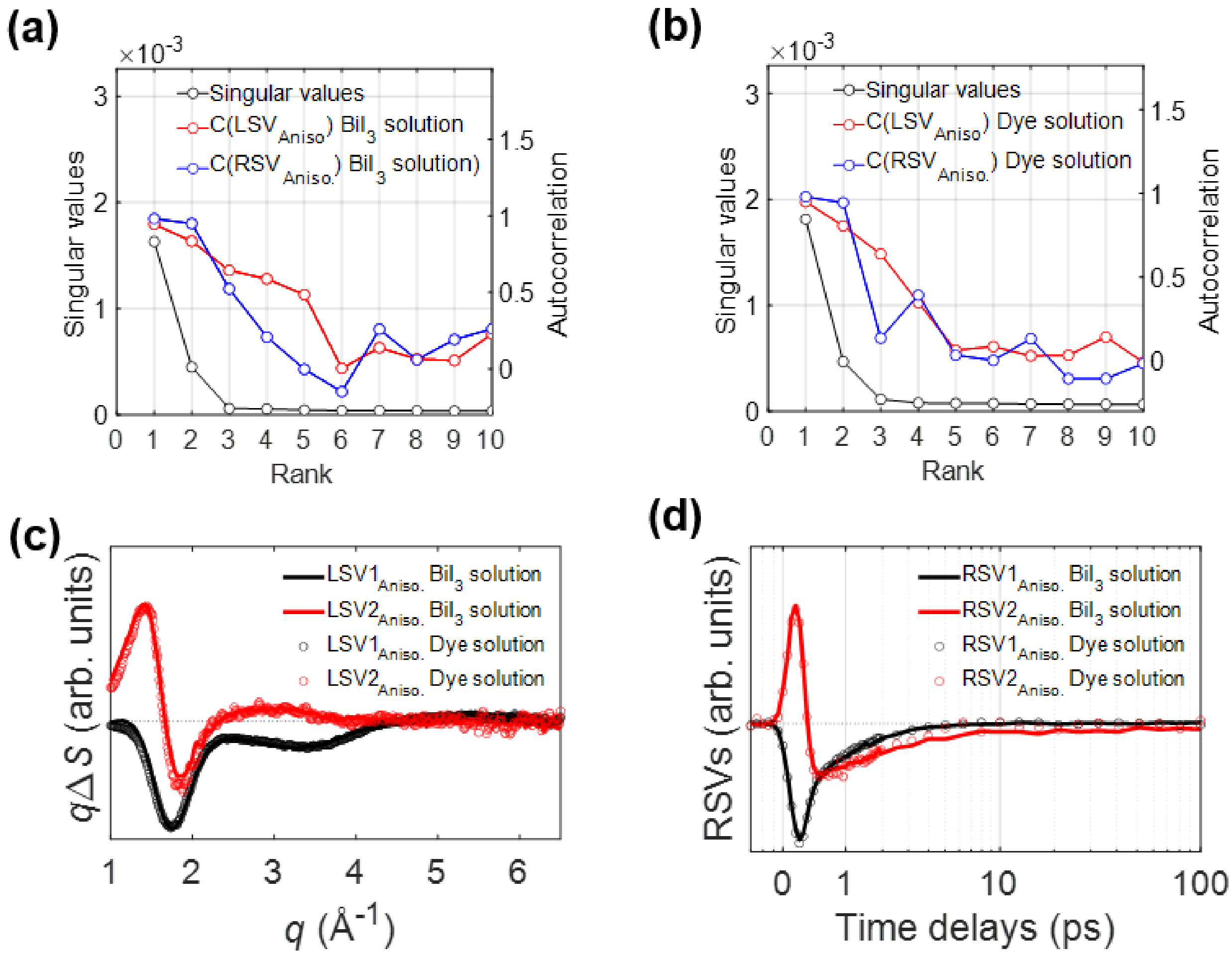

Supplementary Fig. 3. Comparison of the SVD results of the $\Delta S_{aniso}(q, t)$ from the BiI_3 solution and the dye solution. (a, b) The singular values and the autocorrelation values for RSVs and LSVs obtained through SVD of $\Delta S_{aniiso}(q, t)$ of (a) BiI_3 solution and (b) azobenzene dye solution. In both cases, the singular values and autocorrelation values indicate that up to two singular value components are contributing significantly to the signal. (c, d) Plots of the first two (c) left singular vectors (LSVs) and (d) right singular vectors (RSVs) for BiI_3 solution (solid lines) and azobenzene dye solution (circles).

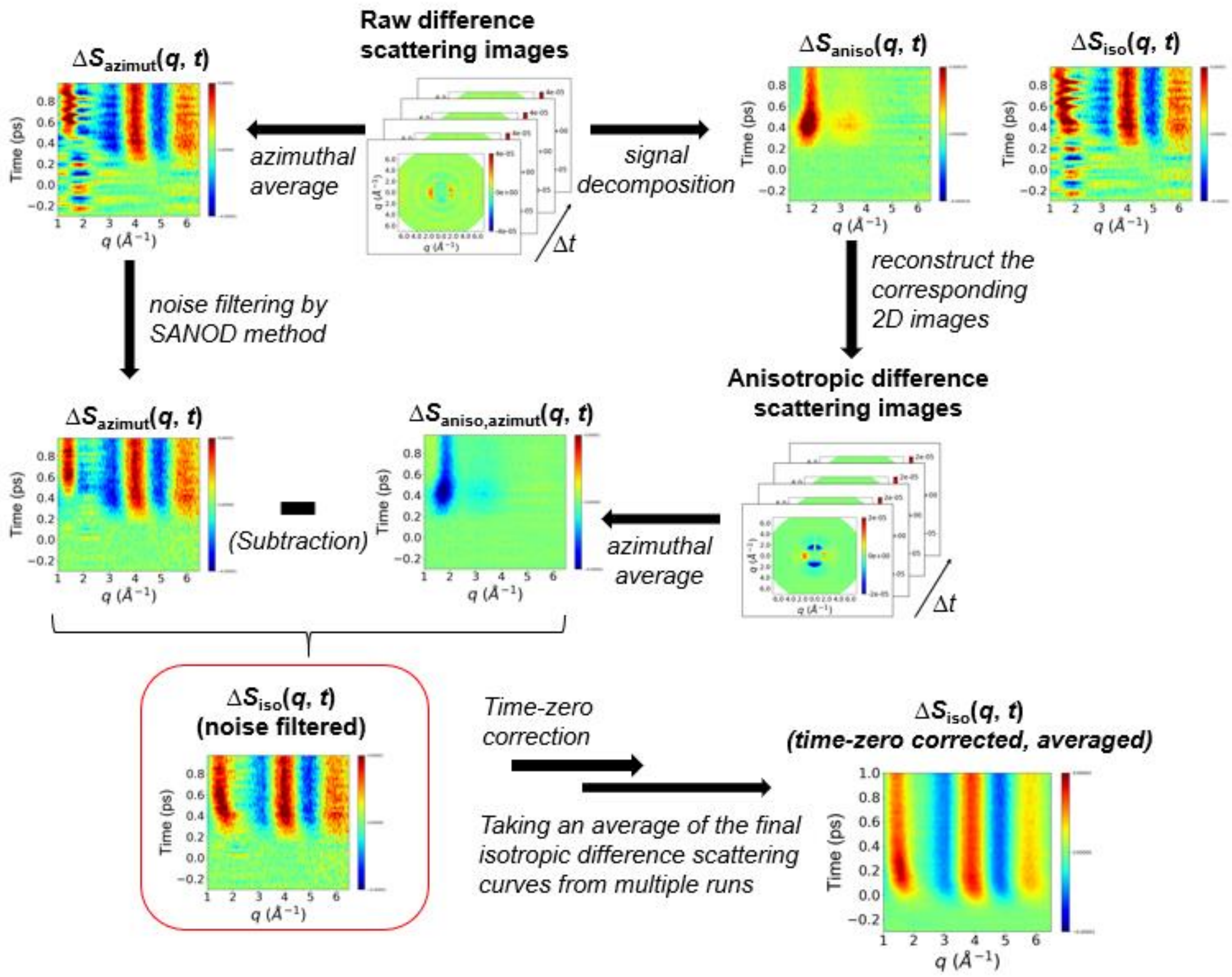

Supplementary Fig. 4. A schematic illustration of the procedures of data processing.

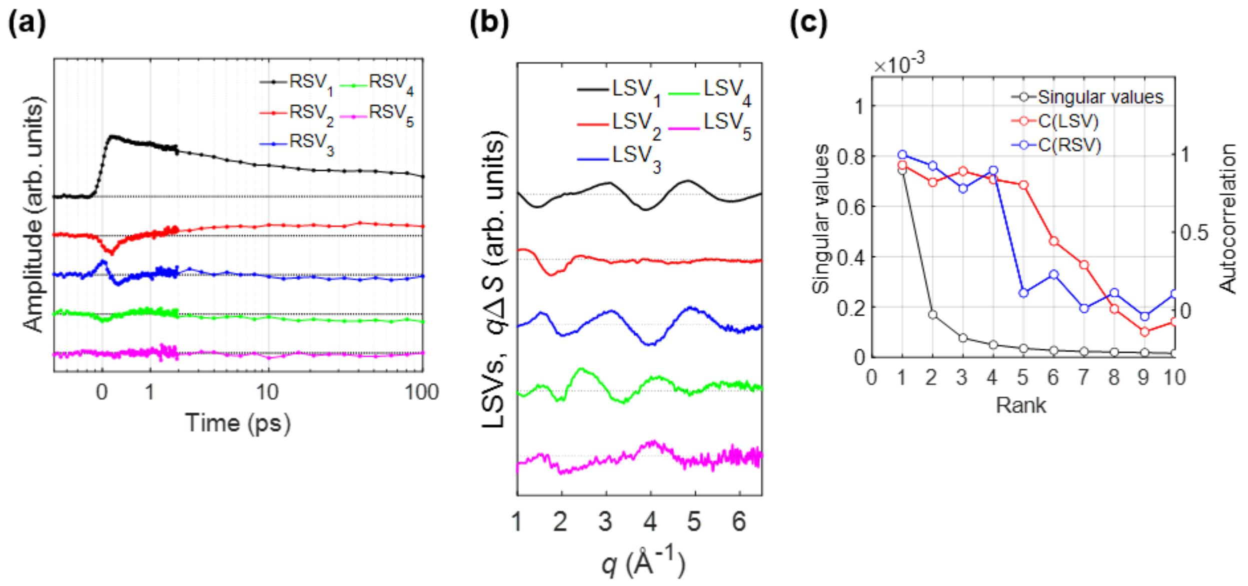

Supplementary Fig. 5. (a) The first five right singular vectors (RSVs) weighted by the corresponding singular values. (b) The first five left singular vectors (LSVs). (c) The singular values, and the autocorrelation values for RSVs and LSVs obtained through SVD of $\Delta S_{iso}(q, t)$ of BiI_3 solution. The singular values and autocorrelation values indicate that up to four singular value components are contributing significantly to the signal.

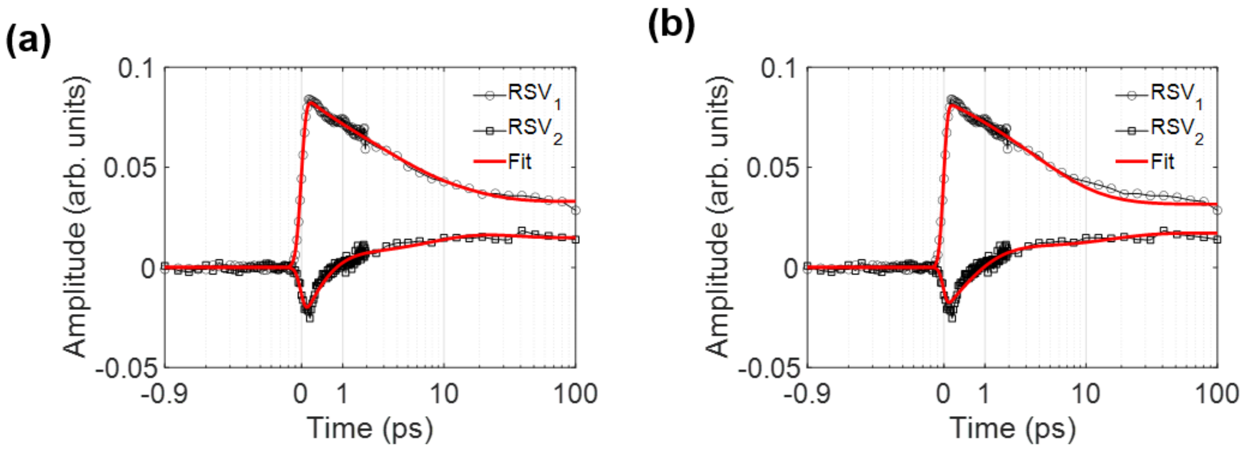

Supplementary Fig. 6. Fits of RSV1 and RSV2 using a sum of (a) four and (b) three exponential functions convoluted with a Gaussian IRF.

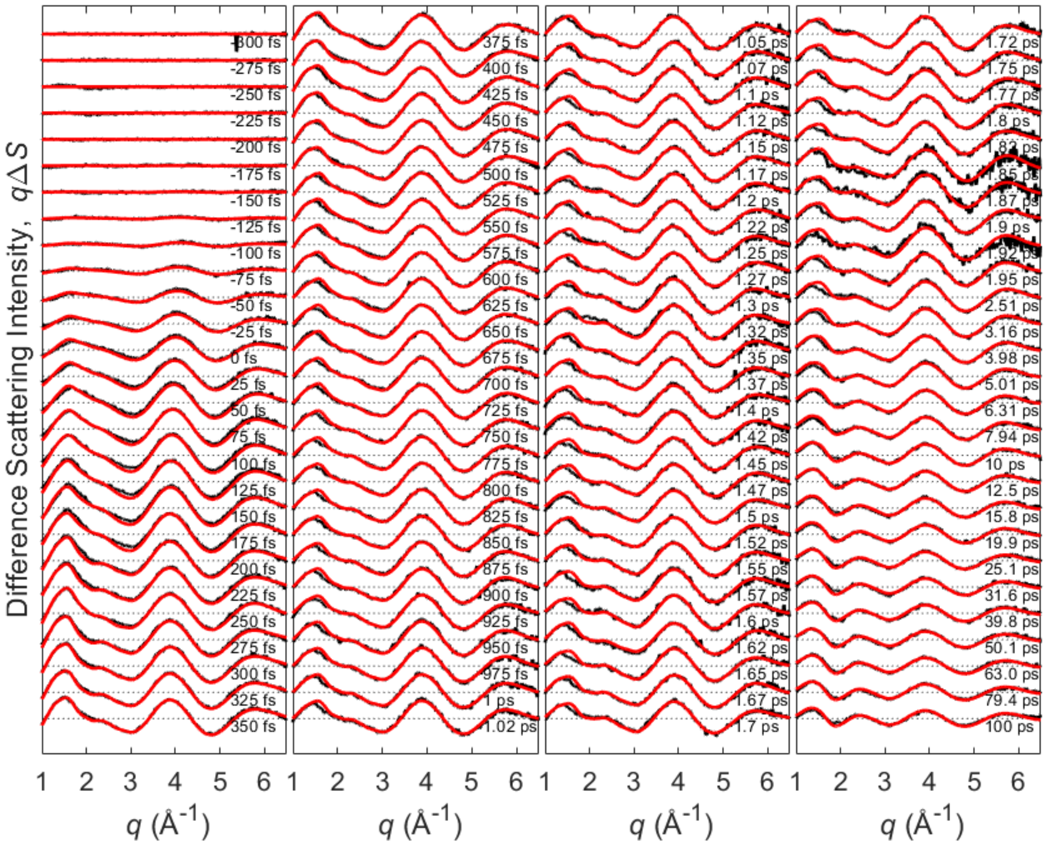

Supplementary Fig. 7. Fit results for the $\Delta S_{iso}(q, t)$ of the BiI_3 solution. Experimental curves and the theoretical curves are shown in black and red lines, respectively.

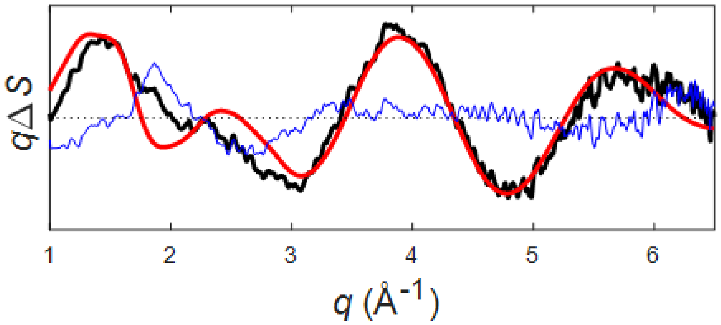

Supplementary Fig. 8. Linear combination fit (red) on ΔS_{iso}'(q, 1 ps) (black) using the scattering curves of (BiI_2 + I) and *iso*-BiI_2-I and the solvent heating curve, $(\partial S(q)/\partial \rho)_T$. The blue line shows the residue obtained by subtracting the fit curve from the experimental scattering curve.

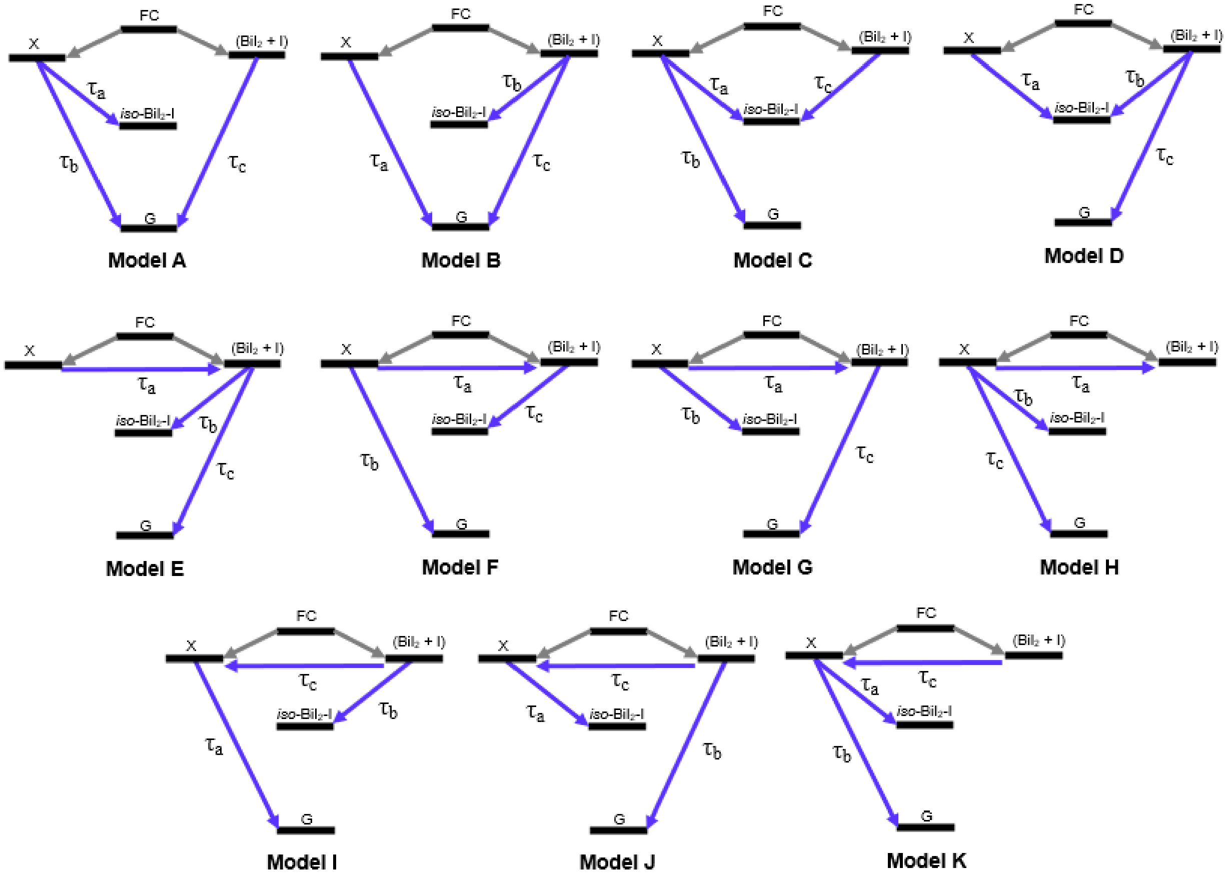

Supplementary Fig. 9. Kinetic model frames used in the global analysis. G and FC denote the BiI_3 at the ground state and Franck-Condon state, respectively.

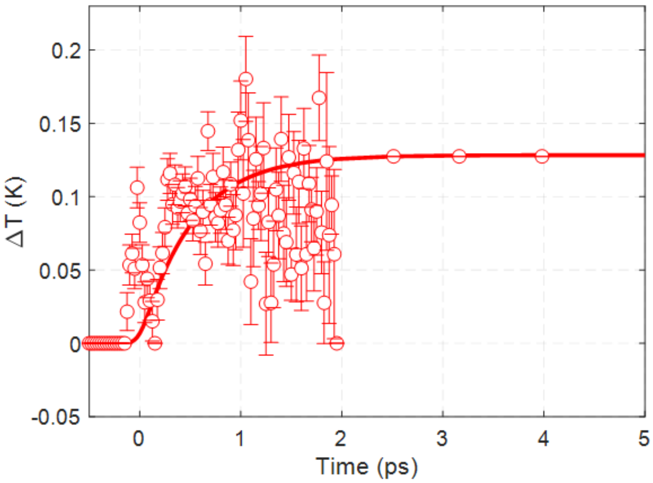

Supplementary Fig. 10. The time evolution of solvent temperature obtained from GFA (solid red line) and linear combination fits (open circle with one-standard-deviation error bars) of $\Delta S_{\mathrm{iso}}'(q, t)$. The solvent heat component in the linear combination fit was used as a free parameter up to 2 ps and then fixed to the global fit result.

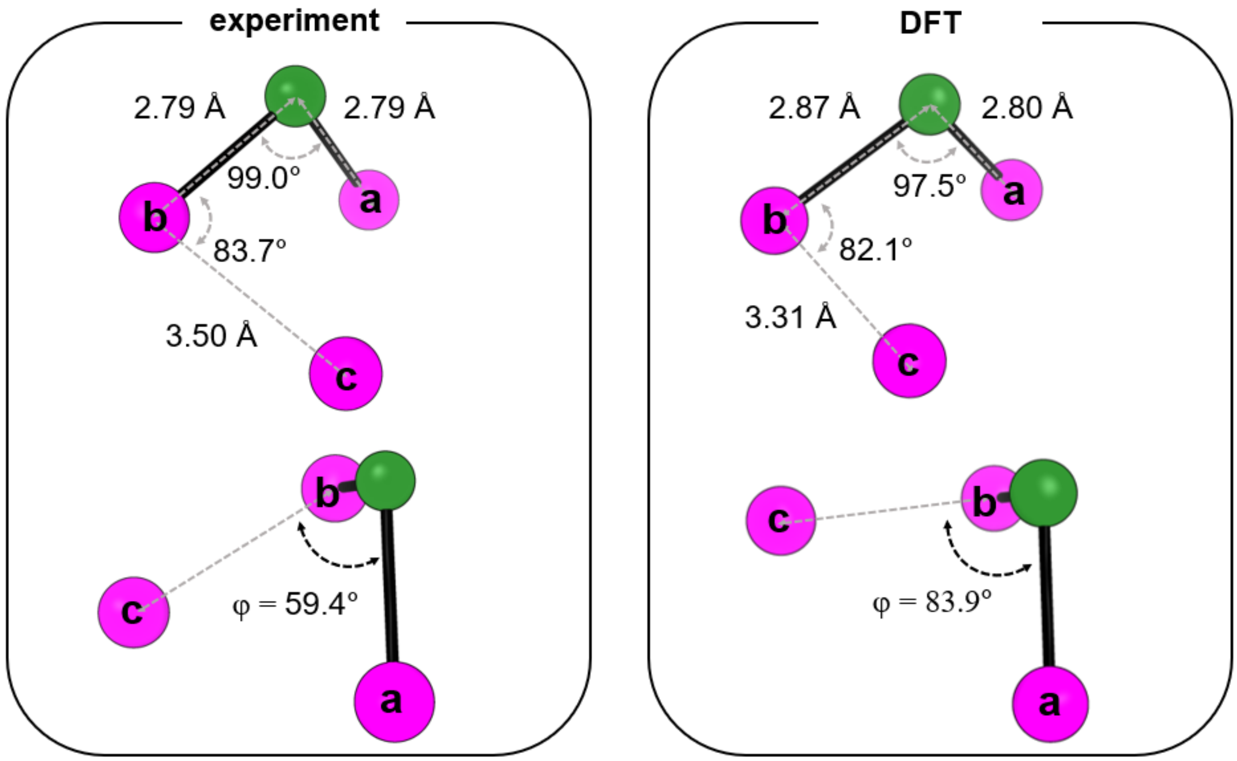

Supplementary Fig. 11. The optimized structure of the early isomer in the global fit analysis (experiment) is compared with the DFT-optimized structure of the early isomer using CAM-B3LYP/dhf-TZVPP. Green and purple spheres stand for Bi and I atoms, respectively, and three I atoms are labeled with a, b, and c.

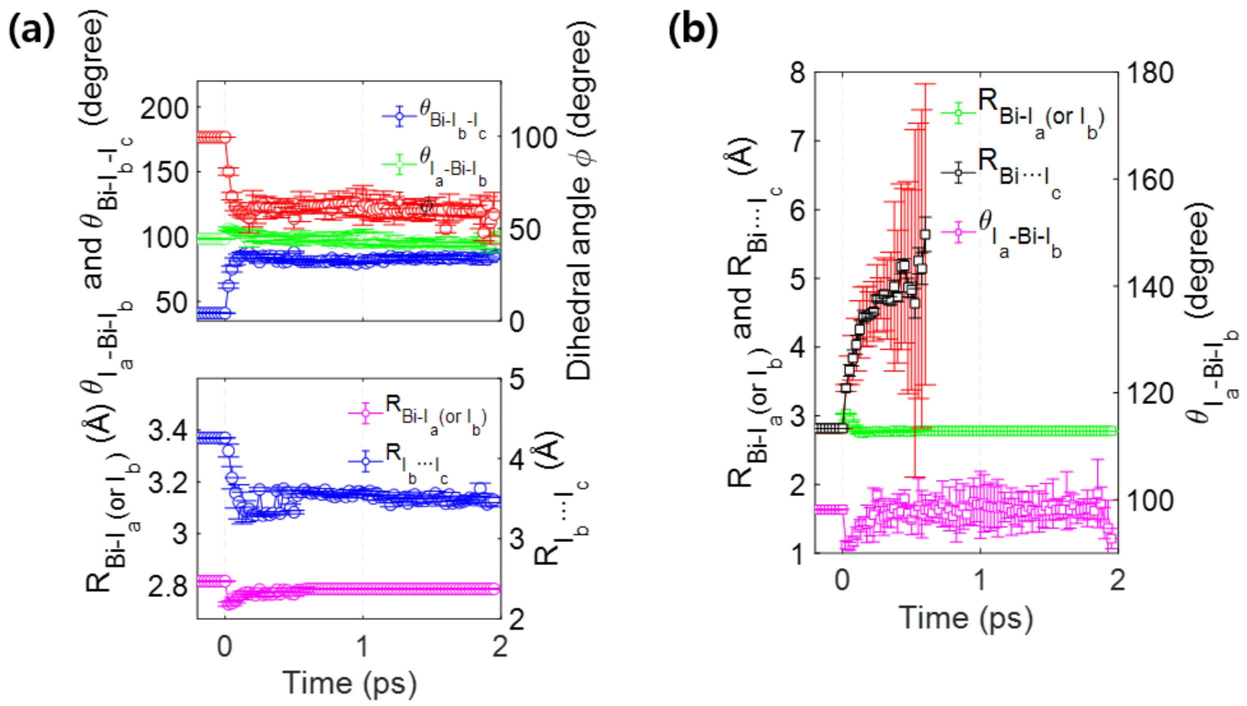

Supplementary Fig. 12. (a) The mean values of the structural parameters for the BiI_3 undergoing isomerization. Angles and distances are shown in the top and bottom panels, respectively. In the top panel, the Bi-I_b-I_c angle, the I_a-Bi-I_b angle, and the I_a-Bi-I_b-I_c dihedral angle (ϕ) are shown in blue, green and red circles, respectively, with the corresponding standard deviations as error bars. In the bottom panel, the Bi-I_a (or I_b) and $I_b \cdots I_c$ distances are shown in magenta and blue circles, respectively, with the corresponding standard deviations as error bars. (b) The mean values of the structural parameters for the dissociating BiI_3. The Bi-I_a (or I_b) bond length, Bi$\cdots I_c$ distances, and I_a-Bi-I_b angle are plotted in green, black, and magenta squares, respectively, with the corresponding standard deviations as error bars. For the Bi$\cdots I_c$ distance, the root-mean-squared distance obtained from the Debye-Waller factor is shown together in red error bars. The error bars of structural parameters in (a) and (b) at time delays earlier than 200 fs are one-standard-deviation error values calculated from the covariance matrix of the coefficients of quartic polynomials used to model the parameters during the structural fit analysis. The error bars of structural parameters in (a) and (b) at time delays later than 175 fs is the averaged one-standard-deviation obtained from the error values of the optimized geometries, which scored less than 1.00001 times the minimum χ^2_{red} (t) obtained from the fit based on ~5000 random structures for each time delay.

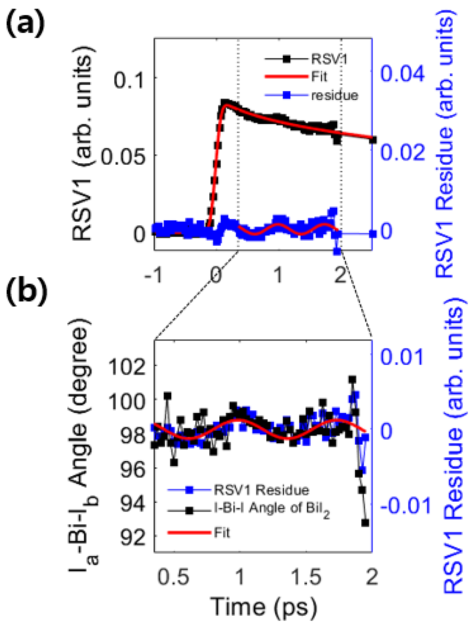

Supplementary Fig. 13. (a) The first right singular vector (RSV1) and the residue obtained by subtracting the exponential fit from the RSV1 are shown in black and blue dots, respectively. Red solid curves on the RSV1 and the residue are the fit curves using exponential and sine functions, respectively. (b) The temporal profile of I_a-Bi-I_b angle of BiI_2 that best represents the oscillating signal in the RSV1 is shown in black dots. The oscillating residue signal and the fit are plotted in blue dots and solid red line together.

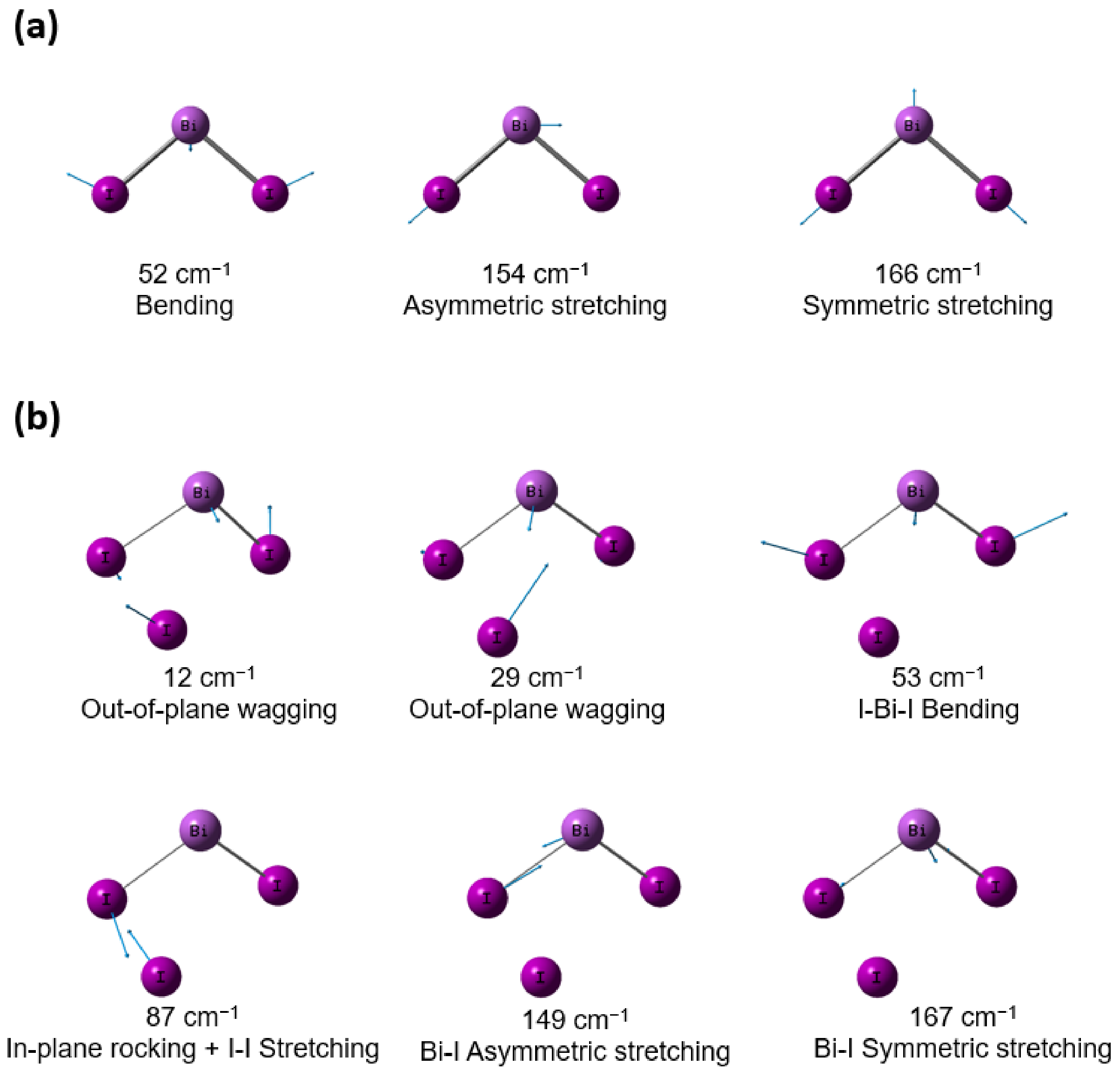

Supplementary Fig. 14. Normal modes of the (a) BiI_2 and (b) early isomer of BiI_3, obtained from CAM-B3LYP/dhf-TZVPP. Displacement vectors of each normal mode are shown in blue arrows.

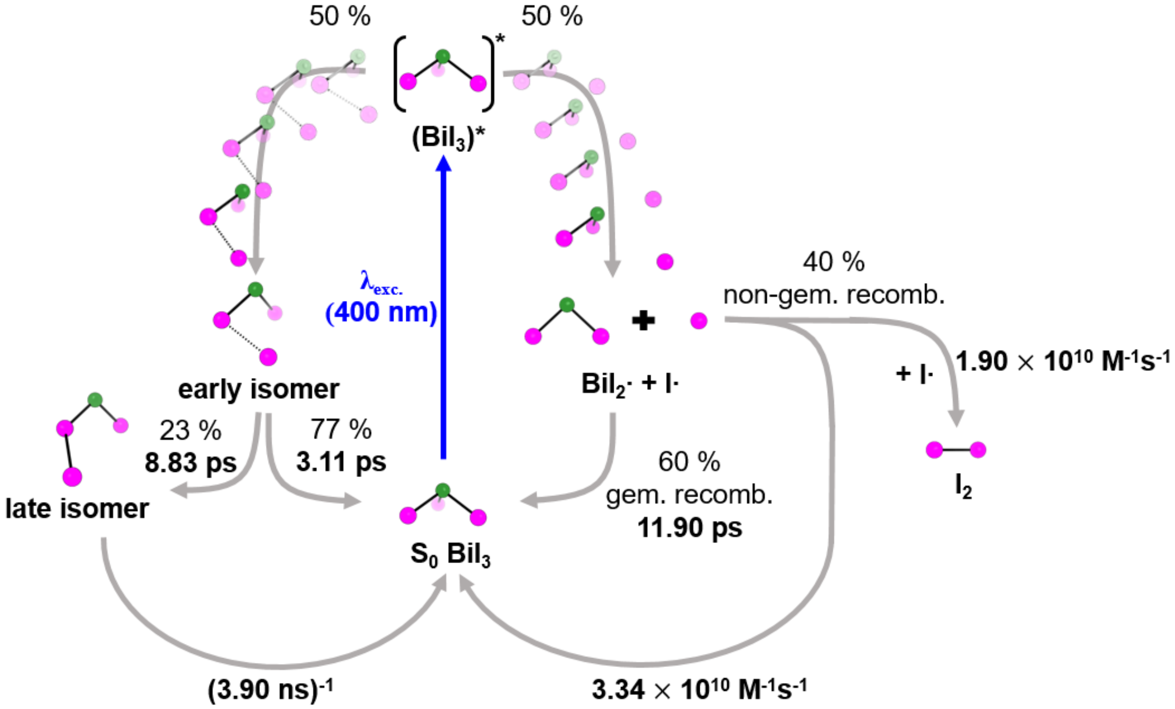

Supplementary Fig. 15. A complete kinetic scheme of the photodynamics of BiI_3 in acetonitrile from 0 fs to 1 μs determined by TRXL.

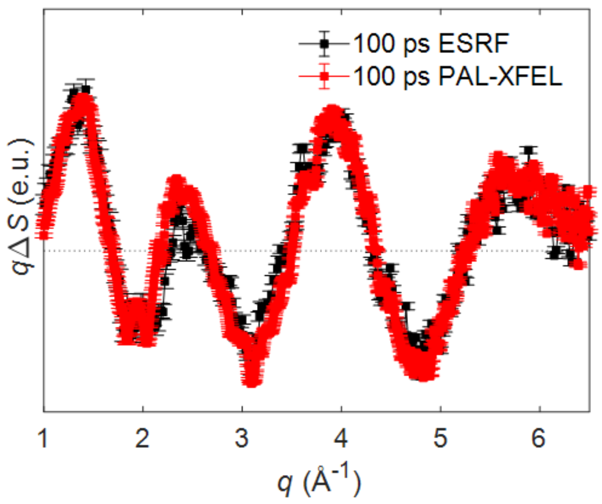

Supplementary Fig. 16. Comparison of the data at a common time delay (100 ps) measured at ESRF and PAL-XFEL. The error bars are one standard-deviation of the mean of the measured signals.

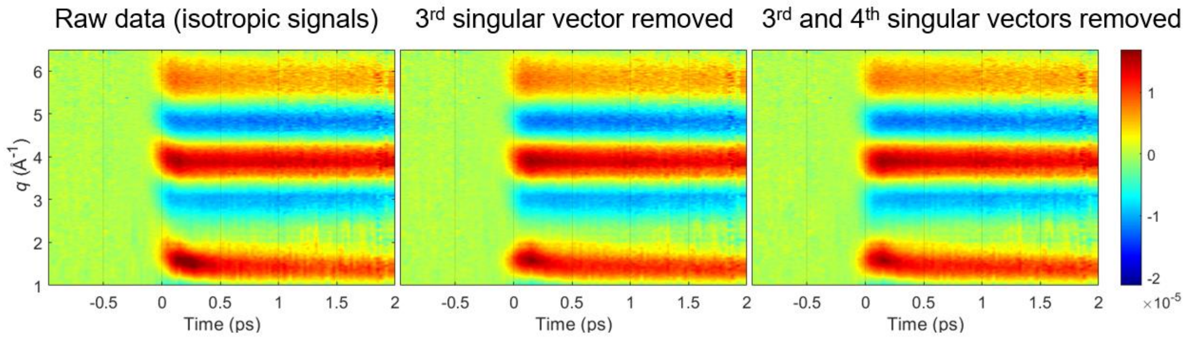

Supplementary Fig. 17. Comparison of raw $\Delta S_{\mathrm{iso}}(q, t)$, SVD-filtered $\Delta S_{\mathrm{iso}}(q, t)$ where the 3rd SV was removed (middle), and SVD-filtered $\Delta S_{\mathrm{iso}}(q, t)$ where the 3rd and 4th SVs were removed (right). Peak shifts in the early time (up to 500 fs) over the entire q-range disappear when both 3rd and 4th singular vectors are filtered out. All plots share a color scale representing the amplitude of the signal in an arbitrary unit.

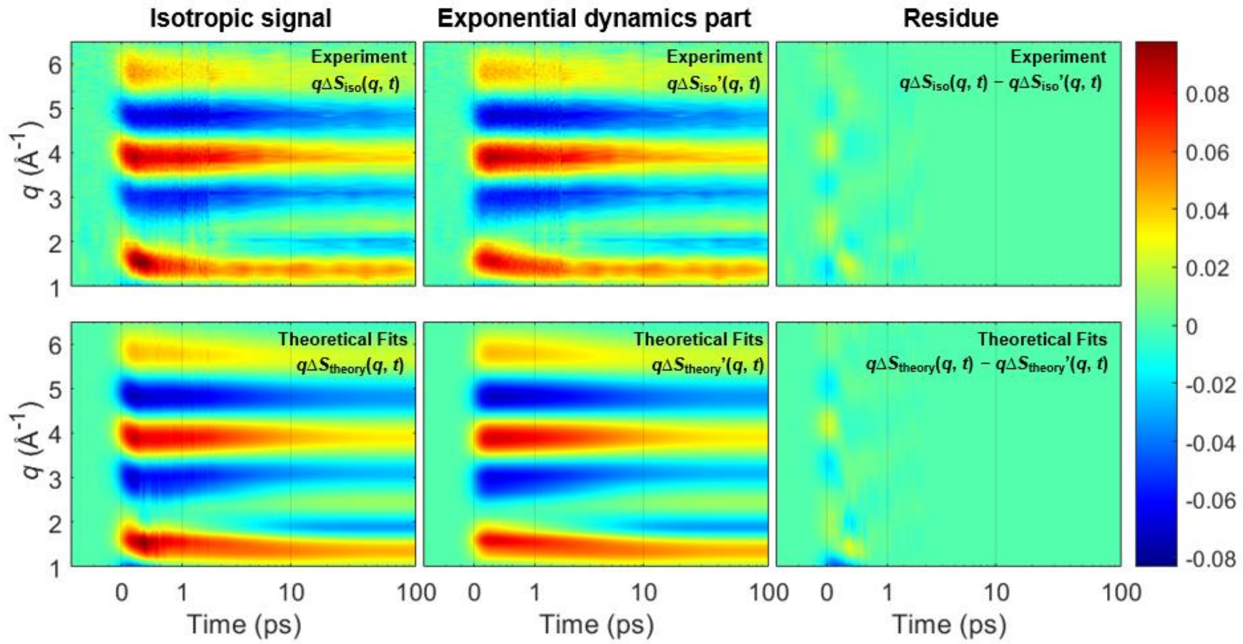

Supplementary Fig. 18. Extraction of the signals for exponential kinetics from the experimental $\Delta S_{iso}(q, t)$ (top panels). The corresponding fit results are shown together in the bottom panels. Residue curves of both experimental and theoretical curves are obtained by subtracting $\Delta S_{iso}'(q, t)$ and $\Delta S_{theory}'(q, t)$ from $\Delta S_{iso}(q, t)$ and $\Delta S_{theory}(q, t)$, respectively. All plots share a color scale representing the amplitude of the signal in absolute electronic units per solvent molecule.

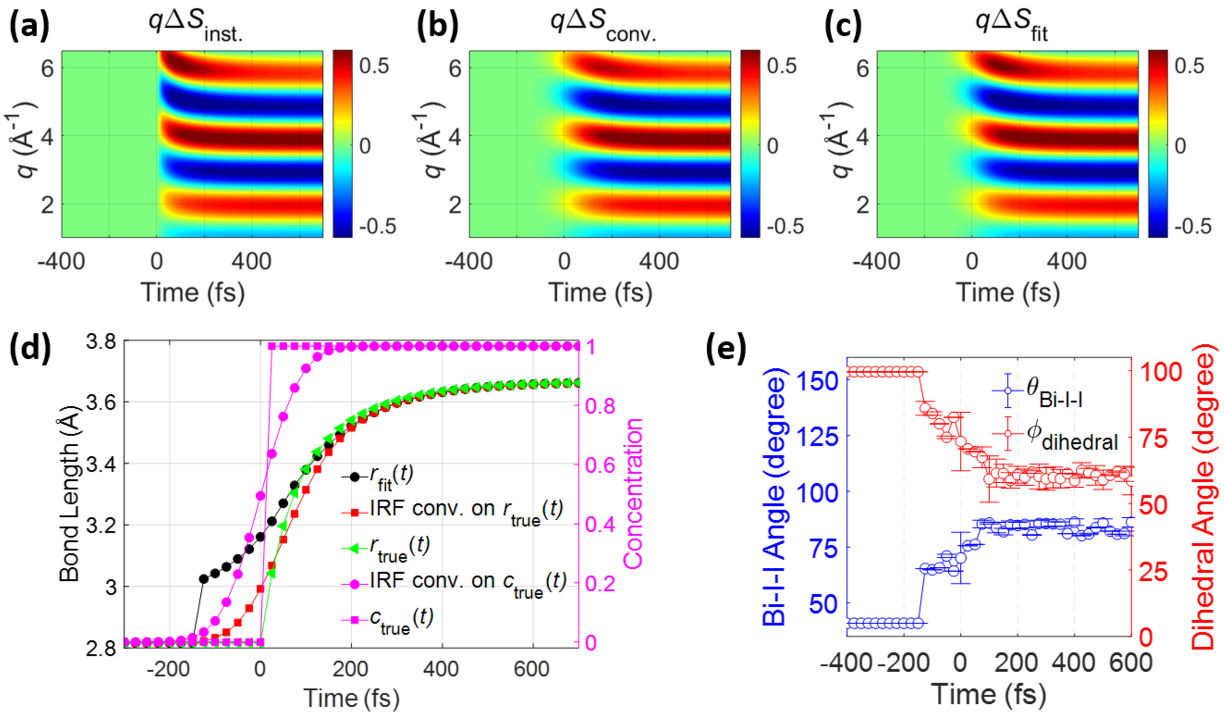

Supplementary Fig. 19. Mock data analysis for testing the effect of the IRF. (a) Instantaneous mock difference scattering signals, $q\Delta S_{\mathrm{inst}}$, of a hypothetical diatomic molecule undergoing a bond elongation from 2.816 Å to 3.616 Å following a sum of two exponential growth functions whose time constants are 50 fs and 150 fs, respectively. (b) IRF-convoluted mock data, $q\Delta S_{\mathrm{conv}}$. (c) Theoretical fit on $q\Delta S_{\mathrm{conv}}$. Color scales in (a), (b), and (c) represent the amplitude of the signal in an arbitrary unit. (d) The temporal profile of the bond length, $r_{\mathrm{true}}(t)$, used to generate $q\Delta S_{\mathrm{inst}}$, the IRF-convoluted $r_{\mathrm{true}}(t)$, and the bond length obtained from the fit on $q\Delta S_{\mathrm{conv}}$, $r_{\mathrm{fit}}(t)$, in green triangles, red squares and black circles, respectively. The temporal profile of the instantaneous excited state concentration of the hypothetical diatomic molecule that was used to construct $q\Delta S_{\mathrm{inst}}$ and the IRF-convoluted excited state concentration that was used to fit the $q\Delta S_{\mathrm{conv.}}$ are shown in magenta squares and circles, respectively. (e) The temporal profiles of the Bi-I_b-I_c angle and I_a-Bi-I_b-I_c dihedral angle of BiI_3, which are obtained from directly fitting $\Delta S_{\mathrm{iso}}(q, t)$ of BiI_3 solution. The error bars are the one-standard-deviation error values obtained from averaging the optimized geometries, which scored less than 1.01 times the minimum $\chi^2_{\mathrm{red}}(t)$ obtained from the fit based on ~1850 random structures for each time delay.

Supplementary Table 1.

Excitation energies (in nm) of BiI_3 calculated by MS-CASPT2+SOC/ANO-RCC-VTZP with the CPCM method. [a]Values in parentheses are oscillator strengths.

Excitation energies[a]	Composition of spin-free states
413 nm (0.18×10^{-2})	S_4(29.0%) T_5(26.7%) T_6(26.2%) S_2(5.0%)
403 nm (0.11×10^{-3})	T_1(45.1%) T_3(29.8%) T_7(6.7%)
402 nm (0.15×10^{-3})	T_1(38.7%) T_2(19.8%) T_4(15.9%) T_3(7.8%)
383 nm (0.33×10^{-3})	T_3(41.3%) T_2(18.9%) T_4(16.0%) S_5(5.8%)

1. References

1. Ko, I. S. et al. Construction and Commissioning of PAL-XFEL Facility. *Appl. Sci.* **7**, doi:Artn 47910.3390/App7050479 (2017).
2. Kang, H. S. et al. Hard X-ray free-electron laser with femtosecond-scale timing jitter. *Nat. Photonics* **11**, 708-713, doi:10.1038/s41566-017-0029-8 (2017).
3. Ki, H. et al. SVD-aided non-orthogonal decomposition (SANOD) method to exploit prior knowledge of spectral components in the analysis of time-resolved data. *Struct. Dyn.* **6**, doi:Artn 02430310.1063/1.5085864 (2019).
4. Lorenz, U., Møller, K. B. & Henriksen, N. E. On the interpretation of time-resolved anisotropic diffraction patterns. *New J. Phys.* **12**, 113022, doi:10.1088/1367-2630/12/11/113022 (2010).
5. Biasin, E. et al. Anisotropy enhanced X-ray scattering from solvated transition metal complexes. *J. Synchrotron Radiat.* **25**, 306-315, doi:10.1107/S1600577517016964 (2018).
6. Choi, E. H. et al. Structural Dynamics of Bismuth Triiodide in Solution Triggered by Photoinduced Ligand-to-Metal Charge Transfer. *J. Phys. Chem. Lett.* **10**, 1279-1285, doi:10.1021/acs.jpclett.9b00365 (2019).
7. James, F. & Roos, M. Minuit - System for Function Minimization and Analysis of Parameter Errors and Correlations. *Comput. Phys. Commun.* **10**, 343-367, doi:Doi 10.1016/0010-4655(75)90039-9 (1975).
8. Kim, T. K. et al. Spatiotemporal Kinetics in Solution Studied by Time-Resolved X-Ray Liquidography (Solution Scattering). *ChemPhysChem* **10**, 1958-1980, doi:10.1002/cphc.200900154 (2009).
9. Refson, K. Moldy: a portable molecular dynamics simulation program for serial and parallel computers. *Comput. Phys. Commun.* **126**, 310-329, doi:Doi 10.1016/S0010-4655(99)00496-8 (2000).
10. Cammarata, M. et al. Impulsive solvent heating probed by picosecond x-ray diffraction. *J. Chem. Phys.* **124**, doi:Artn 12450410.1063/1.2176617 (2006).
11. Kjaer, K. S. et al. Introducing a standard method for experimental determination of the solvent response in laser pump, X-ray probe time-resolved wide-angle X-ray scattering experiments on systems in solution. *Phys. Chem. Chem. Phys.* **15**, 15003-15016, doi:10.1039/c3cp50751c (2013)
