# Supplementary material for: Filming ultrafast roaming-mediated isomerization of bismuth triiodide in solution
Source: Nat Commun. 2021 Aug 5;12:4732. doi: 10.1038/s41467-021-25070-z (PMC8342516; doi:10.1038/s41467-021-25070-z)
Supplement: Supplementary file 3 — Description of Additional Supplementary Files [file 41467_2021_25070_MOESM3_ESM.docx]

# File Name: Supplementary Data 1 Description: Relative *χ*^2^_red_ values for the tested kinetic models. From the four time constants obtained from SVD analysis (τ_1_ = 508 fs, τ_2_ = 3.11 ps, τ_3_ = 8.83 ps and τ_4_ = 11.90 ps), three of the four time constants are chosen and assigned to the solute kinetics based on the eleven kinetic model frames shown in Supplementary Fig. 9. Overall 264 kinetic models (24 kinetic models per each kinetic model frame) are tested in the global fit analysis on Δ*S*_iso_’(*q*, *t*), and the resultant relative *χ*^2^_red_ are tabulated. Designations of the kinetic models are marked in bold. The relative *χ*^2^_red_ for the best kinetic model is 1.000 and marked in red-bold text. The minimum *χ*^2^_red_ was obtained in the kinetic model A3.

**File Name: Supplementary Data 2
Description:** Optimized structural parameters for the early isomer from the global fit analysis based on various kinetic models. The structure of the early isomer was robustly determined regardless of the details of the kinetic model.
